# Testing associations between language use in descriptions of playfulness and age, gender, and self-reported playfulness in German-speaking adults

**DOI:** 10.3389/fpsyg.2022.935009

**Published:** 2022-09-01

**Authors:** Kay Brauer, Rebekka Sendatzki, René T. Proyer

**Affiliations:** Department of Psychology, Martin Luther University Halle-Wittenberg, Halle (Saale), Germany

**Keywords:** adult playfulness, OLIW, LIWC, personality, language

## Abstract

Adult playfulness describes individual differences in (re)framing everyday situations as personally interesting, and/or entertaining, and/or intellectually stimulating. We aimed at extending initial evidence on the interconnectedness between language use and adult playfulness by asking 264 participants (*M* = 26.5 years, *SD* = 9.7; 66.7% women) to provide written descriptions of their understanding of playfulness (mean length: 30.6 words; *SD* = 24.1) and collected self-reports of their playfulness. We used the Linguistic Inquiry and Word Count methodology to quantitatively analyze the language use in these descriptions and tested the associations with individual differences in participants’ age, gender, and playfulness. While higher expressions in all measures of playfulness did go along with writing more content when describing playfulness (*r*s = 0.13 to 0.25), facet-wise analyses revealed differential findings (e.g., intellectual playfulness relates to using words describing cognitive processes); but the effects were small. We found that being a women and younger age were related to writing longer texts (0.13 ≤ *r*s ≤ 0.24), and we discovered additional associations between certain LIWC categories and age and gender. Our study expands the knowledge about adult playfulness and its manifestations in natural language use. We embed our findings into previous research and discuss limitations and potential approaches for replication studies.

## Introduction

Thoughts and emotions are expressed through language, and language use has been identified as a marker of individual differences (Jackson et al., 2022; see also Baumgarten, 1933; Allport and Odbert, 1936). Since Pennebaker and King (1999) introduced automated language analysis with the *Linguistic Inquiry and Word Count* (LIWC) software, research on how personality traits are expressed in language has received robust interest. We aimed at extending the knowledge on the associations between language use and trait playfulness in adults by analyzing textual descriptions of playfulness and age, gender, and self-reported playfulness.

### Adult playfulness

Adult playfulness describes individual differences in how people (re)frame situations so that they are perceived as personally interesting, and/or entertaining, and/or stimulating (Proyer, 2017). The OLIW model is a structural model of playfulness and differentiates among four facets: ***O****ther-directed* (i.e., using playfulness to uplift others or relieve tension in social situations), ***L****ighthearted* (i.e., tendency to view life as a game, to enjoy improvising, and to worry little about the consequences of one’s actions), ***I****ntellectual* (i.e., enjoyment of playing with ideas, complexity, and challenges and problems that require novel approaches), and ***W****himsical* playfulness (i.e., preferences for extraordinary or unusual things and people, appear unconventional to others; Proyer, 2017). The associations between the OLIW facets and age and gender are negligible, although intellectual playfulness is related to higher age and other-directed playfulness goes along with younger age; but effect sizes are small (Proyer, 2017). There is increasing interest in the study of adult playfulness (Bittermann et al., 2021), as research continues to identify its contribution to the understanding of domains such as romantic relationships, creativity, and mental and physical health (e.g., Proyer et al., 2018, 2019; Brauer et al., 2021; Farley et al., 2021).

### Adult playfulness and language

Playfulness and language have been studied with different methodologies. For example, Barnett (2007) tested self- and other ratings toward 42 adjectives (derived from focus groups with young adults) and verbs to identify descriptors that relate to self-reported playfulness (e.g., “active,” “creative,” and “humorous”). Factor analyses of the 15 predictors that discriminated low and high scorers in playfulness suggested a four-dimensional structure.

Proyer (2012a) conducted a text corpus analysis of natural German language, testing the occurrence of the word “play(ful)” (and common inflectional terms), and identified about 15,000 hits. He identified categories describing common content and derived a list of 112 statements (*Playfulness Incidents in Adults*; PIA). The PIA list contained statements that were not covered in established questionnaires on playfulness, showing the importance of learning more about a trait such as playfulness by considering natural language. Further, Proyer asked 240 participants to describe themselves by responding to the PIA list. Factor analyses showed that the 112 statements were best described by seven factors and that some factors (e.g., intellectual playfulness) were underrepresented in existing measures at the time. Proyer (2014a) replicated the study in an independent sample but retrieved only five factors. These, however, widely overlapped with those of the prior study. Further, testing relationships with self-reports of playfulness and humor allowed to discriminate playfulness from theoretically related variables and test the overlap with playfulness. The study of playfulness based on natural language use contributed to understanding the structure of playfulness and its distinction from other constructs.

Gordienko-Mytrofanova et al. (2019) asked participants to provide five words that they associate with the stimulus word “playfulness.” Their psycholinguistic analysis showed that responses could be clustered into 12 categories such as “cheerful course and joyful state,” “intention to attract the attention of the opposite or one’s own sex,” “child-like spontaneity,” “ease,” and “mental activity.” Their findings contributed to a better understanding of what people in Ukraine typically associate with the concept of playfulness and helped to identify semantic components of how people think about playfulness.

An alternative approach to study language use is computerized text analysis with LIWC (Pennebaker and King, 1999). LIWC analyzes text data by matching the words used with an internal dictionary and provides the frequencies of word use for about 90 categories covering formal and grammatical (e.g., first-person singular words; word count) and psychological themes (e.g., words indicating affective and social processes, drives, and personal concerns). Individual differences in age, gender, and personality traits are reflected in language use assessed with LIWC (e.g., Fast and Funder, 2008; Hirsh and Peterson, 2009; see Tausczik and Pennebaker, 2010 for an overview). The LIWC methodology has contributed to identifying language markers that relate to objective outcomes such as synchronicity of couples and application success based on analyses of application letters (Ireland and Pennebaker, 2010; Brandt and Herzberg, 2020).

Proyer and Brauer (2018) used LIWC for testing associations between playfulness and language use based on short written self-descriptions (≤ five sentences). Other-directed playfulness was associated with the usage of first-person plural pronouns (“we”), lighthearted playfulness was related to using fewer words regarding school and achievements, intellectual playfulness was associated with basic linguistic dimensions (words per sentence) and with using more work-related words, and whimsical playfulness went along with higher usage of music-related words, whereas words from the social processes and achievement categories were used less when describing themselves. In addition, Proyer and Brauer showed that playfulness could be judged well from the self-descriptions by independent observers and that judgments were related to the LIWC criteria, suggesting that judges “use” certain linguistic cues for their inferences on playfulness. Taken together, the literature supports the notion that playfulness is expressed in language.

### The present study

Our aim was to expand the knowledge on how playfulness is reflected in language by asking participants to provide descriptions of what they understand as playfulness and using LIWC for text analysis. Prior research has shown that people have a comparatively differentiated understanding of what playfulness is: Proyer (2014b) asked 299 participants to name ways to use playfulness and found that people produced between 0 and 27 functions (*M* = 6.2, *SD* = 3.6, median = 6) that could be clustered into seven categories (e.g., wellbeing, mastery orientations, and relationships; see Barabadi et al., 2022, for a replication). Women reported more functions than men and higher self-reported playfulness related to reporting more functions and greater diversity (i.e., responses covered many of the broader categories). In this study, we followed this rationale and collected data on self-reports of playfulness and textual descriptions of participants’ understanding of playfulness. Using LIWC to analyze the language use in the descriptions systematically allowed us to study the associations between individual differences in language use operationalized by the frequencies in LIWC categories with age, gender, and self-reports of playfulness. In line with findings from the study of lay people (Proyer, 2014b), we expected positive associations between text length (word count) and identifying as a woman (H1) and self-reported playfulness (H2). Considering Proyer and Brauer’s (2018) findings on playfulness-language associations from LIWC analyses of self-descriptions, we also expected that other-directed would relate to using words describing social processes (H3) and first-person plural pronouns (“we,” H4). For whimsical playfulness, we expected to find greater use of words belonging to the LIWC category “leisure” (H5). Finally, we hypothesized associations between playfulness and the use of positive emotions. Playfulness has been linked to the facilitation of positive emotions (e.g., Fredrickson, 2004; Proyer, 2014b). Although this has not been supported in an earlier study in the self-descriptions provided (Proyer and Brauer, 2018), we revisited this hypothesis and expected a positive relationship with the positive emotions LIWC category (H6) for the writing task in this study. We expected to find the typical effect sizes typically reported for personality-LIWC correlations (≈0.23; Hirsh and Peterson, 2009). Finally, LIWC findings on age suggest that higher age relates to using fewer negative and more positive emotion words and fewer self-references (Tausczik and Pennebaker, 2010). However, it is unclear whether this also applies to language use when describing playfulness. Thus, we tested these correlations in an exploratory fashion.

## Methods

### Participants and procedure

Our sample comprised 264 participants [66.7% women, 33.3% men; *M* = 26.5 years (18, 66), *SD* = 9.7]. About two-thirds were students (66.3%), 28.7% were employees, and the remainder (5.0%) were unemployed or retired. We advertised the online study^1^ *via* social media and leaflets in 2019 as “online study on personality and language.” There was no financial compensation for participation, but psychology students could earn course credit. When entering the online study, participants provided informed consent, completed two questionnaires on playfulness, and were asked to freely write up to five sentences about the topic “adult playfulness” (see Supplementary material for full instruction). On average, participants completed the study in 20 min.

Power analyses (G*Power; Faul et al., 2009) showed that our data allowed detecting the average LIWC-personality effect size (ρ = 0.23; Hirsh and Peterson, 2009) with 96% power and 5% type-I error rate (two-tailed tests). Sensitivity power analyses showed that our sample size allowed us to detect ρs ≥ 0.18 with 80% power.

### Instruments

The questionnaires were presented online, and participants gave their responses on a seven-point Likert-type scale (1 = *does not apply at all*, 7 = *applies completely*). Sample items are provided in the Supplementary material.

The five-item *Short Measure of Adult Playfulness* (SMAP; Proyer, 2012b) assesses an easy onset and frequent display of playful behaviors. The SMAP has good psychometric properties (αs ≥ 0.80; test–retest correlation of 0.74 for up to 16-week intervals; α = 0.84 in this study), and there is robust evidence for its validity (Proyer, 2012b).

The *OLIW-Playfulness Questionnaire* (Proyer, 2017) assesses four facets of adult playfulness (other-directed, lighthearted, intellectual, and whimsical) with seven items each. The OLIW demonstrates good psychometric properties (e.g., test–retest reliabilities ≥ 0.67 up to a 3-month interval, internal consistencies ≥ 0.66) and validity (Proyer, 2017). The internal consistencies in this study aligned with prior research, with α = 0.66/0.76/0.54/0.71 (other-directed, lighthearted, intellectual, and whimsical playfulness).

We used Meier et al.’s (2019) German-language version of the *Linguistic Inquiry and Word Count* software (LIWC; Pennebaker et al., 2015). LIWC scans text data, matches the words with an internal dictionary, and provides the frequency of word usage in each of about 90 pre-set categories that cover basic linguistic variables (e.g., pronouns), psychological constructs (e.g., *affect*), and personal concerns (e.g., *work* and *leisure*). There is robust evidence for the reliability and validity of the German-language version of LIWC (Meier et al., 2019). As recommended by Meier et al. we spellchecked the texts before computing the LIWC analysis. We excluded categories that refer to spoken conversations (e.g., *fillers*). The word “Verspieltheit” (playfulness) is not in the German LIWC dictionary.

## Results

The means and *SD*s in the playfulness measures aligned with prior studies of German samples (see Supplementary material for all coefficients). Intellectual playfulness was related to age (*r* = 0.18, *p* = 0.003), and men were higher in the SMAP, lighthearted, and whimsical playfulness (small effect sizes; 0.13 ≤ *r*s ≤ 0.14, Hedges’ *g*s between –0.06 and 0.31; see Supplementary material).

The textual descriptions of playfulness contained between 1 and 197 words^2^ (*M* = 30.6, *SD* = 24.1, median = 25.0), and LIWC recognized on average 86.3% (*SD* = 11.0%, median = 88.4%) of the textual information. We examined the associations between age, gender, playfulness, and LIWC frequencies by computing bivariate correlations (see Supplementary material for full results). For the playfulness-LIWC associations, we computed partial correlations, controlling for age and gender. In line with conventions (e.g., Hirsh and Peterson, 2009), we corrected the correlation coefficients for the LIWC’s reliability (0.59).

### Gender

In line with H1, women (coded = 1; men coded as 2) wrote slightly more about playfulness than men (word count: *r* = |0.13|, *p* = 0.032; words per sentence: *r* = |0.24|, *p* < 0.001). Exploratory analyses showed that women used more pronouns (*r* = |0.27|, *p* < 0.001), particularly personal pronouns. In contrast, men used more female references (*r* = 0.20) and words concerning work (*r* = 0.21, *p*s ≤ 0.001) and leisure (*r* = 0.18, *p* = 0.003). Controlling for playfulness (SMAP) did not affect our findings (Δ*r* ≤ 0.03).

### Age

Higher age was related to lower word count (*r* = –0.19) and words per sentence (*r* = –0.30, *p*s ≤ 0.003), to using more first-person singular references (*r* = 0.19, *p* = 0.002), and there was a small association with using third-person singular references (*r* = 0.12, *p* = 0.048) when describing playfulness. In line with the latter, age was related to describing social processes (*r* = 0.13, *p* = 0.003). In line with findings on age and language use (Tausczik and Pennebaker, 2010), we found minor associations with using positive emotion words (*r* = 0.13, *p* = 0.040), but no relation to using negative emotion words (*r* = -0.07, *p* = 0.082).^3^ Controlling for playfulness did not change the findings (Δ*r* < 0.01).

### Playfulness

In line with H2, self-reported playfulness was related positively to writing more about playfulness (word count), with *r*s = 0.24 (SMAP), 0.25 (other-directed), 0.17 (lighthearted), 0.18 (intellectual; *p*s < 0.003), and 0.13 (whimsical; *p* = 0.033). Further, those higher in other-directed, intellectual, and whimsical playfulness wrote longer sentences (*r*s between 0.14 and 0.18, *p*s ≤ 0.021). Against expectations, other-directed playfulness did not relate to using more words describing social processes (H3; *r* = –0.05, *p* = 0.418) and first-person plural pronouns (H4; *r* = –0.04, *p* = 0.518). Similarly, we did not find support for a relationship between whimsical playfulness and using more leisure words (H5; *r* = –0.01, *p* = 0.869). Finally, we tested whether playfulness was related to using positive emotion words. Against expectations, we found negative associations between positive-emotion word usage and global playfulness (*r* = –0.15, *p* = 0.014) and lighthearted playfulness (*r* = –0.17, *p* = 0.006).

Exploratory analyses showed some notable findings that should be highlighted: global playfulness related to using fewer words with future time orientation (*r* = –0.22, *p* < 0.001) and more health-related words (*r* = 0.16, *p* = 0.011). Greater expressions in lighthearted playfulness were related to using fewer anxiety-related words (*r* = –0.20) and fewer words relating to risk (*r* = –0.20, *p*s = 0.001). Intellectual playfulness was related to words indicating cognitive processes, particularly from the category of insight (*r* = 0.20, *p* < 0.001).

## Discussion

We aimed at expanding prior research on the interplay between playfulness and language by examining the language use in textual descriptions of playfulness. Contrary to prior research testing existing language corpora (e.g., Proyer, 2014a) or single-word associates in response to “playfulness” as a stimulus word (Gordienko-Mytrofanova et al., 2019), we collected more comprehensive descriptions of playfulness (i.e., full sentences) and used quantitative text analyses (LIWC; Pennebaker and King, 1999) to examine how individual differences in age, gender, and self-reported playfulness relate to language use when writing about playfulness. Overall, our findings in terms of effect sizes were in the range typically found in LIWC studies (e.g., Hirsh and Peterson, 2009). In line with Proyer’s (2014b) study on perceptions of functions of playfulness, participants differed in how much content they produced in the writing task (word count). As expected, those with higher scores in playfulness produced more words overall and longer sentences. This aligns well with Proyer’s finding that people higher in playfulness reported more functions of how they can use playfulness in everyday life. Furthermore, we found that women provided longer texts on playfulness, again, in line with the finding that women reported more perceived functions of playfulness.

Given that Newman et al.’s (2008) meta-analysis of gender differences in LIWC variables did not indicate that women would generally produce more words than men, our finding seems not to be based on generalized gender differences in writing styles. However, it must be noted that the length of the texts is a metric that does not consider the quality of the texts, and, thus, we cannot conclude that longer texts on playfulness provided more heterogeneity or saturation in the perceived functions and reported incidents of playfulness. While our findings supported hypotheses 1 and 2, we did not find support for hypotheses 3–5 that were based on prior findings of LIWC-playfulness correlations (Proyer and Brauer, 2018) and the notion that playfulness relates to using positive emotions (H6). This might be partly explained by differences in the writing task. Earlier research has shown that language use also depends on the content people are asked to write about (e.g., Pasupathi, 2007). While Proyer and Brauer (2018) asked participants to provide *general self-descriptions* and Gordienko-Mytrofanova et al. (2019) asked participants to provide five *words* that they associate with the stimulus word “playfulness,” we specifically asked our participants to write openly about *playfulness*, irrespective of whether they described personal experiences, associations, or generalized convictions and perceptions of playfulness. Potentially, this also shows a shift in the perception of play and playfulness in the scientific literature: while earlier research has highlighted the importance of play and playfulness for joy, fun, and entertainment, newer research has an additional focus on intellectual types of playfulness or how playfulness can be used for innovativeness. Hence, participants may not have thought only about the fun aspects of playfulness but also about how they can capitalize on their playfulness in different areas of their lives. This is also in line with Gordienko-Mytrofanova et al.’s findings showing the broad variety of what people understand as playful(ness).

To our knowledge, no study has thus far examined language use in connection to playfulness in naturalistic contexts. Analyzing everyday speech assessed with electronically activated recorders (Mehl et al., 2001) or natural writings (e.g., blog and diary entries, letters, or emails) could allow capturing language use that is not restricted by instructions or boundaries of a formal writing task and further examine whether the hypothesized relations between playfulness and language use exist.

Exploratory analyses await replication and extension in future research. For example, self-reports of intellectual playfulness went along with using descriptors of cognitive processes, particularly the subcategory of insight (e.g., “think,” “know,” and “consider”). This fits well into the conceptualization of intellectual playfulness (i.e., preferences for complexity and liking to think about problems; Proyer, 2017) and its associations with creative thinking and curiosity (e.g., Proyer et al., 2019). Those high in lighthearted playfulness show low inclinations to worry about future consequences (Proyer, 2017), and this manifests in negative relations to neuroticism and negative affectivity (Proyer et al., 2020). Accordingly, self-reported lighthearted playfulness in our study went along with the use of fewer anxiety-related words (e.g., “worried” and “nervous”) and fewer risk-related words (e.g., “danger,” “doubt”). Finally, expressions in a global measure of playfulness were associated with using health-related words, which comports with previous studies showing that playfulness is associated with engaging in physical activity and greater physical and mental health (e.g., Proyer et al., 2018; Gordienko-Mytrofanova et al., 2019; Farley et al., 2021) and people describing that playfulness contributes “to be active” and “to motivate myself and others” (e.g., Proyer, 2014b; Gordienko-Mytrofanova et al., 2019). Also, we found that global playfulness is related to using more future tense words (e.g., “will,” “gonna”), which has been previously connected to goal orientation (Tausczik and Pennebaker, 2010) and might also be indicative of using playfulness by means of mastery orientation and challenging future tasks (Proyer, 2014b). While these findings should not be overinterpreted, they provide initial evidence for the reflection of playfulness in language use and might be starting points for further research.

In line with the literature (Tausczik and Pennebaker, 2010), women used more social references than men. It is notable that men used more work- and leisure-related words, which could indicate that they show greater inclinations for using playfulness in these contexts (cf. Proyer, 2014b). Also, men used greater references to women, and following the well-supported notion of playfulness contributing to facilitating and maintaining close relationships (e.g., Proyer, 2014b; Gordienko-Mytrofanova et al., 2019; Brauer et al., 2021), men’s descriptions of playfulness containing more references to women could reflect using playfulness to engage with the opposite gender.^4^

As expected, higher age was related to using more positive-emotion words and a greater focus on social aspects (i.e., social processes and third-person singular words; e.g., “she”). Contrary to typical findings, older participants used more, instead of fewer, first-person singular words (“I,” “me”) when describing playfulness. Since this type of self-reference predicts numerous outcomes (e.g., status, honesty, and depression; Tausczik and Pennebaker, 2010), we cannot put forward a single interpretation, but argue that this finding might be specific to writing about playfulness considering the writing task and that correlations were not affected when controlling for self-reported playfulness. Future research examining the role of age, playfulness, and their interconnectedness with language is desirable, especially using longitudinal research that allows testing within-person change in language use over time. Our cross-sectional data cannot address whether associations are based on cohort effects or typical age developments.

### Limitations and future directions

Our study has several limitations. First, we only collected self-reports of playfulness, which might be affected by biases. There is robust convergence between self- and other reports of playfulness (e.g., Proyer, 2017), and using additional information from peer ratings or behavioral data of playfulness would supplement the self-reports and reduce biases (Campbell and Fiske, 1959). Further, playfulness can be accurately inferred from self-descriptions (Proyer and Brauer, 2018), and future research might test if the same is true for written accounts of playfulness. Using external judgments also helps to understand implicit theories of playfulness: for example, observers inferred that people were more playful when they used positive-emotion words, irrespective of the missing relation between self-reported playfulness and the use of positive-emotion words (Proyer and Brauer, 2018).

While our research is of descriptive nature, future research might use multivariate approaches to predict external criteria (e.g., language style matching as a function of playfulness; Ireland and Pennebaker, 2010). Second, we only tested German speakers, which limits the generalizability of our findings, and an open question is how the findings would translate to other language contexts. For example, Pang and Proyer (2018) discussed that playfulness is more negatively perceived (i.e., as the opposite of seriousness) in Eastern countries as compared to Western countries and found that Chinese samples showed lower expressions of playfulness compared to German participants. Moreover, they discussed that a term that precisely reflects “playfulness” is missing in the Chinese language and that the term “play” is avoided when describing activities (e.g., using “performing on the piano” instead of “playing the piano;” see also Yu et al., 2007; Shen et al., 2021). We expect that this would also affect formal and content-wise aspects of writings about playfulness, and we cannot assume cross-cultural invariance of our findings. Third, our writing task restricted the lengths of texts to make the entries more comparable, thus setting a boundary on the text lengths. Fourth, it must be noted that LIWC is based on quantitative (frequency) analyses of language use, and although the dictionary covers words that relate to psychologically relevant variables, it is limited in capturing holistic meanings and complex syntactic structures. However, despite this limitation, we argue that the findings aligned well with prior research studying functions of playfulness as judged by humans (Proyer, 2014b) and the nomological net of playfulness. Fifth, future research might extend the study of gender differences in textual descriptions of playfulness, for example, by comparing playfulness-LIWC correlations among men and women in a well-powered sample to examine the invariance of findings across gender. Finally, although the LIWC dictionary coverage was high, the LIWC dictionary is not comprehensive, and future studies should re-examine the data upon the release of a revised German LIWC dictionary to categorize the complete textual input.

In conclusion, our findings contribute to the knowledge on the interplay of individual differences in language use and expressions in self-reported adult playfulness, age, and gender. Future research should examine more naturalistic language, using multiple sources of information (e.g., peers and behavioral data) and examine the cross-language invariance of our findings. Also, extension to dyadic contexts by analyzing reciprocal communication and dyadic indexes (Ireland and Pennebaker, 2010) is desirable. Finally, LIWC detects changes in writing styles after interventions (Jackson et al., 2022). Proyer et al. (2021) introduced three 1-week trainings (e.g., counting playful experiences across the day) that increased expressions of playfulness up to 12 weeks post-intervention. We expect that systematic changes in playfulness go along with changes in language use pre-to-post training, which could contribute to identifying linguistic reflections of playfulness.

## Data availability statement

The datasets and syntaxes presented in this study can be found in online repositories. The names of the repository/repositories and accession number(s) can be found below: https://osf.io/fkhqn/.

## Ethics statement

Ethical review and approval was not required for the study on human participants in accordance with the local legislation and institutional requirements. The patients/participants provided their written informed consent to participate in this study.

## Author contributions

KB, RS, and RP: conceptualization, roles, and writing—original draft and review and editing. RP: data collection. KB and RS: formal analysis. KB: methodology. All authors contributed to the article and approved the submitted version.

## Footnotes

1
www.soscisurvey.de

2 The one-word statement was “Sport” (English: “sports”), and there was also a two-word statement which read “Herumkichern, Infantil” (English: “giggling around, infantile”). There was no indication of careless responding by these participants.

3 On the descriptive level, age was related to using more health-related (*r* = 0.15, *p* = 0.012) and achievement-related words (*r* = 0.18, *p* = 0.003), and related negatively to describing cognitive processes (*r* = –0.15, *p* = 0.013) and drive for risk (*r* = –0.16, *p* = 0.008). Concerning time perspectives, age relates to focusing on the past (*r* = 0.20, *p* < 0.001) and present (*r* = 0.15, *p* = 0.018). The findings should not be overinterpreted and await replication.

4 We have not collected data on participants’ sexuality. Thus, our speculation assumes heterosexual mating. Of course, future research must test whether our speculation holds in groups of homosexuals (i.e., men using more male references; women using more female references) and bisexuals to support this notion.
