# Supplementary material for: Testing associations between language use in descriptions of playfulness and age, gender, and self-reported playfulness in German-speaking adults
Source: Front Psychol. 2022 Sep 1;13:935009. doi: 10.3389/fpsyg.2022.935009 (PMC9477000; doi:10.3389/fpsyg.2022.935009)
Supplement: Supplementary file 1 [file Table_1.DOCX]

**ESM A**

*Instruction for the Writing Task*

German: “Bitte formulieren Sie zunächst bis zu fünf Sätze, die Ihnen zum Thema Verspieltheit im Erwachsenenalter in den Sinn kommen. Denken Sie zum Beispiel daran, ob Sie selbst verspielte Dinge in Ihrem Leben machen. Die Länge der Sätze bleibt dabei ganz Ihnen überlassen.“

Tentative English translation: „Please write up to five sentences on the topic of adult playfulness that come to mind. For example, think about the playful things you do in your life. The length of the sentences is entirely up to you.“

**ESM B**

*Sample Items of the Self-Report Questionnaires of Playfulness*

| Scale | Sample Item |
| --- | --- |
| SMAP | “I am a playful person.” |
| *OLIW Questionnaire* |  |
| Other-directed | “I can use my playfulness to bring joy to other people or cheer them up” |
| Lighthearted | “Many people take their lives too seriously; when things don’t work you just have to improvise” |
| Intellectual | ”If I want to develop a new idea further and think about it, I like to do this in a playful manner” |
| Whimsical | “I have the reputation of being somewhat unusual or flamboyant” |

*Note.* SMAP = Short Measure of Adult Playfulness (Proyer, 2012).

**ESM C**

*Descriptive Statistics and Correlations with Age and Gender for the Playfulness Scales*

|  | *M* | *SD* | Age | Gender |
| --- | --- | --- | --- | --- |
| SMAP | 4.78 | 1.20 | .01 | .14* |
| OLIW |  |  |  |  |
| Other-directed | 5.22 | 0.93 | -.06 | .01 |
| Lighthearted | 4.05 | 1.03 | .10 | .14* |
| Intellectual | 4.21 | 0.86 | .18** | .08 |
| Whimsical | 4.22 | 1.00 | .08 | .13* |

*Note*. *N* = 264. SMAP = Short Measure of Adult Playfulness. Gender was coded as 1 = women and 2 = men. **p* < .05. ***p*<.01. Two-tailed.

**ESM D**

*Associations Between Age, Gender, Playfulness, and Language Use as Measured by Linguistic Inquiry and Word Count (LIWC)*

|  |  |  |  | Playfulness | | | | |
| --- | --- | --- | --- | --- | --- | --- | --- | --- |
|  | Age | Gender |  | SMAP | OTD | LTD | INT | WHI |
| Word Count | -.19** | -.13* |  | .24*** | .25*** | .17** | .18** | .13* |
| **Summary variables** |  |  |  |  |  |  |  |  |
| Analytic Thinking | -.01 | .13* |  | .04 | .04 | -.10 | .01 | -.02 |
| Clout | .07 | .10 |  | -.07 | -.03 | -.04 | -.07 | -.05 |
| Authentic | .07 | -.10 |  | .11 | .07 | .01 | .08 | -.05 |
| Emotional tone | .08 | -.02 |  | .12* | .09 | .14* | .11 | .06 |
| Words/sentence | -.30*** | -.24*** |  | .12 | .14* | .08 | .15* | .18** |
| **Linguistic dimensions** |  |  |  |  |  |  |  |  |
| Total function words | .01 | -.15** |  | .18** | .08 | .13* | .07 | .03 |
| Total pronouns | .16** | -.27*** |  | .10 | .03 | .05 | .05 | -.05 |
| Personal pronouns | .19** | -.18** |  | .13* | .07 | .00 | .05 | .08 |
| 1st pers singular | .19** | -.11* |  | .13* | .06 | -.04 | .04 | .10 |
| 1st pers plural | .00 | -.13* |  | .04 | -.04 | -.06 | -.16* | -.09 |
| 2nd person | -.02 | -.06 |  | .04 | .03 | .04 | .05 | .07 |
| 2nd pers singular | .00 | -.07 |  | .03 | .06 | .03 | .05 | .07 |
| 2nd pers plural | -.10 | .06 |  | .05 | -.15* | .03 | -.03 | .02 |
| 3rd person | .12* | -.01 |  | .01 | .06 | .07 | .07 | .04 |
| 3rd pers singular | .12* | .00 |  | .01 | .06 | .07 | .06 | .04 |
| 3rd pers plural | .00 | .07 |  | -.10 | -.07 | .06 | .03 | .08 |
| Impersonal pronouns | -.06 | -.15** |  | .02 | -.10 | .04 | -.03 | -.16* |
| Articles | .10 | .05 |  | .10 | .08 | .09 | .07 | -.10 |
| Prepositions | -.08 | -.04 |  | .06 | -.01 | .00 | .04 | .05 |
| Auxiliary verbs | .09 | .08 |  | .02 | -.03 | .12 | .07 | .10 |
| Common Adverbs | -.16** | -.02 |  | -.01 | .04 | .02 | .06 | .00 |
| Conjunctions | -.08 | -.02 |  | -.09 | -.02 | .09 | .04 | .10 |
| Negations | -.08 | -.09 |  | -.09 | -.18** | .02 | -.06 | .06 |
| **Psychological processes** |  |  |  |  |  |  |  |  |
| Affective processes | .11* | .02 |  | -.17** | -.09 | -.16** | .00 | -.04 |
| Positive emotion | .13* | .04 |  | -.15* | -.07 | -.17** | .03 | -.03 |
| Negative emotion | -.07 | -.03 |  | -.06 | -.04 | .01 | -.11 | -.04 |
| Anxiety | -.12* | -.07 |  | -.09 | -.04 | -.20** | .02 | -.09 |
| Anger | -.02 | -.01 |  | .08 | .11 | .09 | -.10 | .11 |
| Sadness | -.07 | -.06 |  | .00 | .01 | .02 | -.05 | .03 |
| **Social processes** | .13* | -.02 |  | -.01 | -.05 | .02 | -.05 | .06 |
| Family | .07 | -.05 |  | -.03 | -.01 | -.09 | -.02 | .07 |
| Friends | -.05 | -.04 |  | .00 | -.01 | -.11 | -.14* | .08 |
| Female references | .17** | .20** |  | .04 | .08 | .12 | .05 | .17** |
| Male references | -.13* | -.07 |  | -.02 | -.05 | -.11 | -.06 | .07 |
| **Cognitive processes** | -.15** | -.09 |  | -.04 | -.04 | .11 | .15* | .04 |
| Insight | .05 | -.10* |  | .08 | .06 | .03 | .20** | -.03 |
| Causation | .09 | -.08 |  | -.04 | .02 | .07 | .08 | .12 |
| Discrepancy | -.13* | .08 |  | .02 | -.08 | .12 | .00 | -.01 |
| Tentative | -.14* | .03 |  | -.09 | -.05 | .12 | .01 | .03 |
| Certainty | -.07 | -.09 |  | .06 | .06 | .06 | .08 | .07 |
| Differentiation | -.19** | -.01 |  | .00 | -.08 | .16* | .03 | .04 |
| **Perceptual processes** | -.05 | .00 |  | .05 | .03 | .06 | .05 | .00 |
| See | -.12* | .15** |  | .08 | -.03 | .08 | -.01 | -.07 |
| Hear | .13* | -.08 |  | .05 | .14* | .07 | .10 | .03 |
| Feel | .06 | -.07 |  | -.10 | -.08 | -.14* | -.06 | -.09 |
| **Biological processes** | .14* | .05 |  | .04 | .04 | -.01 | .01 | -.08 |
| Body | .05 | -.02 |  | -.01 | .03 | -.18** | -.10 | -.09 |
| Health | .15** | .09 |  | .16* | .12* | -.01 | .06 | .00 |
| Sexual | -.12* | .03 |  | -.02 | -.11 | .12 | -.07 | -.04 |
| Ingestion | .01 | -.07 |  | .09 | .04 | .07 | .10 | .16** |
| **Drives** | .07 | -.04 |  | .06 | .09 | .00 | .06 | .10 |
| Affiliation | -.01 | -.05 |  | .06 | .05 | -.08 | -.08 | .06 |
| Achievement | .18** | .01 |  | .01 | .04 | .04 | .04 | .06 |
| Power | -.01 | -.01 |  | .04 | .07 | .06 | .17** | .04 |
| Reward | -.01 | -.07 |  | .11 | .06 | .13* | .16** | .05 |
| Risk | -.16** | .03 |  | .04 | -.02 | -.20** | -.02 | .02 |
| **Time orientations** |  |  |  |  |  |  |  |  |
| Past focus | .20*** | .01 |  | -.05 | -.12 | .12 | .15* | -.02 |
| Present focus | .15** | -.07 |  | .03 | .06 | .10 | .09 | -.03 |
| Future focus | -.05 | .01 |  | -.22*** | -.14* | -.09 | -.01 | -.07 |
| **Relativity** | .04 | .09 |  | .00 | -.07 | -.06 | -.04 | -.13* |
| Motion | .20** | .16** |  | -.19** | -.10 | -.14* | -.08 | -.19** |
| Space | -.05 | -.03 |  | .15* | .04 | .06 | .00 | .01 |
| Time | -.01 | .05 |  | -.04 | -.05 | -.07 | .03 | -.07 |
| **Personal concerns** |  |  |  |  |  |  |  |  |
| Work | -.02 | .21*** |  | .12* | -.03 | .05 | .07 | -.15* |
| Leisure | .05 | .18** |  | -.11 | -.05 | -.09 | -.01 | -.01 |
| Home | -.10 | -.07 |  | .10 | .11 | .02 | .05 | .09 |
| Money | .10* | .11* |  | .09 | .02 | .11 | .08 | .01 |
| Religion | .05 | .13* |  | .01 | .06 | .12 | .00 | -.13* |
| Death | .05 | -.03 |  | .13* | .13* | .03 | .04 | .11 |

*Note. N* = 264. Gender was coded as 1 = women and 2 = men. SMAP = Short Measure of Playfulness. OTD = Other-directed playfulness. LTD = Lighthearted playfulness. INT = Intellectual playfulness. WHI = Whimsical playfulness. **p*<.05. ***p*<.01. ****p*<.001. Two-tailed.
